# Analysis of Users’ Perception of Home Care Provided by the Family Health Strategy Team: A Quali–Quantitative Approach

**DOI:** 10.3390/healthcare12222210

**Published:** 2024-11-06

**Authors:** José Victor Marconato, Luís Eduardo Genaro, Aylton Valsecki Junior, Fernanda Lopez Rosell

**Affiliations:** 1 School of Medicine, San Francisco University, Bragança Paulista 12.916-900, SP, Brazil; jose.marconato@mail.usf.edu.br; 2 Postgraduate Program in Collective Health in Dentistry, School of Dentistry, São Paulo State University, Araçatuba 16.015-050, SP, Brazil; luis.genaro@unesp.br; 3 Department of Community Dentistry, School of Dentistry, São Paulo State University, Araraquara 14.801-903, SP, Brazil; aylton.valsecki-junior@unesp.br

**Keywords:** home care services, qualitative research, discourse of the collective subject

## Abstract

Background/Objective: Home care involves actions and services aimed at promoting health. Despite being an important strategy for humanized care, strengthening bonds, and improving access, there is a lack of scientific evidence regarding users’ perceptions of home care. The objective of this study was to analyze the perception of users regarding the home care provided by the Family Health Strategy team. Methodology: A descriptive, quali–quantitative approach was used, with a sample of 60 patients who required home care. Interviews were conducted using a semi-structured guide, and the analysis applied the Collective Subject Discourse. Results: Most participants were female (60%) and aged between 71 and 85 years (45%). The duration of home care varied, with 41.6% receiving care for 13 to 24 months. The majority of participants (61.6%) received home care weekly, while 33.4% were visited every 15 days, and 5% monthly. The most present professionals were community health agents (45%), followed by nursing assistants (18.3%), nurses (15%), and doctors (15%). Dentists and physiotherapists made fewer visits (3.3%). Regarding the care received, 36.6% valued the continuity of treatment, 31.6% were satisfied with the quality, and 25% highlighted the humanization of care. Only 6.6% desired more frequent visits. Conclusions: The analysis of perceptions revealed that participants valued the continuity of treatment and the quality of care received. Home visits were predominantly carried out by community health agents.

## 1. Introduction

The Family Health Strategy consists of a set of actions combined in primary care, aligned with the principles of the Unified Health System, which are based on prevention, promotion, and the recovery of users’ health in a continuous and comprehensive manner [1,2,3,4,5]. It is not limited to the health sector but is also integrated with different sectors to address the determinants and conditions of the health–disease process, grounded in continuous care for the population [1].

In this context, home care emerges as an extension of the Family Health Strategy’s actions, consisting of a set of services and interventions aimed at promoting health, preventing, treating diseases, and providing rehabilitation directly at the user’s home. Home care is characterized by strengthening the bond and humanization of care [6,7,8,9], ensuring continuity of care, improving the patients’ quality of life, and enhancing caregiver satisfaction. Additionally, it contributes to the reduction of emergency room visits, hospitalizations, and readmissions [10,11,12,13,14,15,16,17].

This type of home-based care is operationalized through visits by Family Health Strategy teams to users’ homes, aiming to understand the family dynamics and identify the critical points of the reality experienced by these families. Based on this diagnosis, it is possible to plan adequate and effective health actions, aiming at the recovery and well-being of the assisted individuals [7,18,19].

In home care, the basic principles of the Unified Health System are respected, such as universality—providing care to users who cannot travel to the health unit for treatment; comprehensiveness—offering services to users at all three levels of care; and equity—addressing the needs of those who require health assistance the most. Emphasis is placed on providing humane, qualified, and individualized care [20]. Thus, all these principles strengthen the bond between the population and the health service [7,21].

Although it is a strategy that offers potential benefits for patients and promotes the humanized training of professionals [22], gaps still exist in its development, such as the lack of planning [23], absence of discussions after activities, discontinuity of the process, insufficient technical knowledge, and low problem-solving capacity [3]. Planning for home care activities is still deficient, requiring prior organization, the identification of the target population, and an understanding of epidemiological characteristics [23,24]. It is essential for professionals to have access to patients’ medical records in advance, always respecting the care time, informing patients about the visit schedule, and clarifying its purpose to avoid possible conflicts [24].

Additionally, the connection with the family may be hindered as professionals need to balance this activity with demands from the Basic Health Unit. It is important to highlight that the Oral Health Team makes few visits, and when they do, it is mostly at the family’s request, often due to the patient’s pain, which characterizes an alienated work process based on individual clinical treatment, and is exclusionary and technical [3,25].

This study aims to analyze the perception of users who require home care, an important topic portrayed by current studies [26,27,28,29,30]. However, the literature shows that the methodology proposed in this project has not yet been applied, making the development of complementary studies important.

By understanding the participants’ perceptions of home care, we gain a deeper insight into the demands of this group of patients. Additionally, this research can play a role in highlighting the need for adjustments in the assistance programs offered by family health units, aiming to improve the quality of care. The analysis of patients’ perceptions and opinions can offer valuable insights into areas that require adjustments to better meet the needs and expectations of patients receiving home care.

## 2. Materials and Methods

### 2.1. Study Design

This is a descriptive and qualitative study aimed at investigating users’ perceptions of home care within primary health care.

### 2.2. Sample Selection

The sample was selected non-probabilistically, through purposeful sampling, and the data saturation method was used to determine the point at which information collection becomes redundant, meaning when the themes and categories discussed in the interviews begin to repeat and no new relevant data are obtained [31]. The sample consisted of elderly individuals who required home care (n = 60).

### 2.3. Setting

The research was conducted in Itatiba, São Paulo, a medium-sized city with an estimated population of around 120,858 inhabitants, according to IBGE estimates in 2019.

### 2.4. Ethical Considerations

This study was approved by the Research Ethics Committee of the School of Dentistry of Araraquara, São Paulo State University (UNESP) (CAAE: 69122923.6.0000.5416, Opinion Number: 6.773.591), and all participants signed the informed consent form. This study followed the guidelines of the COnsolidated criteria for REporting Qualitative research (COREQ) checklist and the guidelines established by the Equator Network (Enhancing the QUAlity and Transparency Of Health Research). This study adhered to the established reporting standards to ensure the quality and transparency of the research conducted.

To ensure ethical integrity, participants were identified in the results as “p” followed by a cardinal number.

### 2.5. Interview Procedure

The interviews were conducted individually and in person, and audio was recorded. To ensure a suitable environment and minimize interference, they were conducted in the nurses’ rooms at each health unit. The interviewees had no prior knowledge of the interviewer, avoiding potential conflicts of interest. The adopted method favored direct interaction, allowing interviewees the freedom to express their opinions spontaneously and in detail [31,32].

### 2.6. Data Collection

An open-ended and objective question guide was used, as recommended by Lefevre [32]. The questionnaire was pre-tested in a pilot study and applied by a single, properly trained interviewer. The interviews were conducted between July and October 2023, and the recordings were transferred to a computer for transcription and subsequent analysis.

### 2.7. Data Analysis

Data analysis was performed using the qualitative–quantitative technique Discourse of the Collective Subject [33], with the assistance of Qualiquantisoft^®^ software version 1.3.c, which is freely available. According to Lefevre [32], the Discourse of the Collective Subject technique is based on Social Representation theory, which allows synthesizing shared ideas within a group into a single representative discourse.

Initially, Key Expressions were identified, which are significant excerpts from the individual speeches that capture the essence of the expressed content. These expressions were carefully selected to eliminate superfluous information and focus on what is essential. From these Key Expressions, Central Ideas were identified, which accurately summarize what each individual expressed about the topic. Similar Central Ideas were grouped to create response categories, with which synthesis discourses were developed in the first person singular, representing collective thought [32,33,34].

### 2.8. Quantitative Analysis

In addition to the qualitative analysis, a quantitative analysis of the data was performed using descriptive statistics to identify the relative frequency of responses within the created categories.

## 3. Results

The majority of participants are female (60.0%), while men represent 40.0%. The predominant age group is between 71 and 85 years old (45.0%), followed by those over 86 years old (35.0%), with the smallest proportion of individuals between 65 and 70 years old (20.0%). Regarding marital status, most interviewees are married (43.4%) or in a stable union (30.0%), with a smaller percentage of widowed (13.3%), divorced (10.0%), and single individuals (3.33%). In terms of education level, the majority have incomplete primary education (60.0%), while 15.0% have completed primary education, 8.3% have no formal education, and only 5.0% have incomplete secondary education. Most participants belong to economic class D/E (90.0%).

Regarding the duration of home care, 41.6% of participants have been receiving care for 13 to 24 months, 23.3% for 25 to 36 months, 21.6% for up to 12 months, and 13.3% for 37 to 48 months. The main health conditions that justify home care include chronic obstructive pulmonary disease (35.0%), heart failure (25.0%), neoplasia (15.0%), stroke (16.6%), and arthritis/osteoarthritis/rheumatism (23.3%), with some participants having more than one clinical condition. 

The interviews were transcribed and analyzed using the qualitative–quantitative approach of Collective Subject Discourse.

Below is a descriptive presentation of the data from Table 1, which revealed different categories and subcategories.

### 3.1. Frequency of Home Care 

The frequency of home care varies according to the severity of the patients’ health conditions. The majority of participants, representing 61.6% of responses, receive weekly care. They describe that healthcare professionals visit them regularly, weekly, to provide care, check their condition, and offer guidance on medication and self-care. Here are some statements:

*“Every week, they stop by to see how I’m doing and make sure I’m following the treatment properly”. (p23) Another participant shared, “They visit regularly to monitor my condition and remind me to take my medication on time”. (p47) A similar sentiment was expressed by someone else: “Once a week, they check in on me and help with anything I need, especially my medication”. (p12) Others reported, “They also come to check my blood pressure and ask if I have any concerns about my health”. (p59) and, “Their weekly visits ensure that I’m taking care of myself and staying on top of my treatment plan”. (p34)*

Around 33.4% of interviewees reported receiving home care once every 15 days. These patients noted that healthcare professionals make regular visits every two weeks, providing ongoing support and consistently checking their health status. Some participants said:

*“They stop by every two weeks to check on how I’m doing and if I need any help; additionally, every 15 days, they visit to make sure everything is going smoothly with my health”. (p8) “Moreover, at least twice a month, they come to see if I have any issues or concerns”. (p45) “Furthermore, they visit every two weeks to monitor my progress and ensure I’m following my treatment plan”. (p18) “Finally, once every 15 days, they check in to ask if everything is fine and if there’s anything I need”. (p30)*

A smaller portion, representing 5.0% of participants, mentioned receiving home care once a month. They explained that after recovering from a more serious condition, the visits were reduced to once a month. Now, in better health, these visits have become less frequent.

*“Since my recovery, they visit my home once a month; when I was in worse shape, their visits were more frequent”. (p53) “Now that I’m feeling better, the doctor drops by about once a month, compared to the weekly visits I used to have”. (p9) “I receive home visits every month now that I’m stable; previously, they came by much more often when my health was at risk”. (p36) “The healthcare team checks in on me about once a month these days; back when I was really ill, they were here much more frequently”. (p19) “Every month, they stop by to see how I’m doing; during my hospitalization, they came regularly, but now that I’m better, it’s less frequent”. (p16)*

This analysis demonstrates an adaptation in the frequency of home care based on the clinical evolution of the patients. Most receive weekly care, followed by visits every 15 days, and finally, a smaller proportion receives monthly care.

### 3.2. Which Professional Makes the Most Home Visits

The research data reveal the distribution of healthcare professionals involved in home care, as reported by the interviewees. A descriptive analysis of the data shows that community health agents are the most numerous category, totaling 27 professionals, representing 45.0% of the total. Interviewees’ statements:

*“The health agent is the one who visits us the most; she always makes sure we understand how to take our medication properly”. (p4) “Every week, the health agents stop by to check if we need anything from the health center, including asking if we have enough supplies at home”. (p26) “The agent who comes to our house regularly is very helpful; she clarifies any doubts I have about my medication”. (p11) “Weekly visits from the health agents ensure that we have everything we need from the health center, and they even check if our pantry is stocked”. (p7) “The health agent frequently visits us; she is always ready to explain how to manage my treatment effectively”. (p38)*

Next, nursing technicians stand out, with 11 professionals reported, corresponding to 18.3% of the total. Nurses also have a significant presence, with nine professionals, or 15.0% of the total, demonstrating their relevance in home care. Statements:

*“The individuals from the health post that visit are primarily nurses, who play a crucial role in my care”. (p29) “Typically, a nurse visits my home to check that all aspects of my health are well managed”. (p21) “Most of the visitors from the health center are nurses, ensuring I receive the support I need”. (p14) “A nurse regularly comes to my house to monitor my health and provide any necessary guidance”. (p22) “The nurses from the health post are the ones who come here most often, helping me stay on track with my health”. (p5)*

Doctors have a representation equivalent to that of nurses, also totaling nine professionals, which corresponds to 15.0% of the total. This highlights the importance of these professionals in providing medical care at home.

*“The doctor who frequently visits my home makes sure that all my health concerns are addressed”. (p50) “I have a doctor who regularly comes to check on my well-being and ensures that everything is in order”. (p31) “The doctor visits me often to evaluate my condition and make any necessary adjustments to my medication”. (p60) “I appreciate that the doctor comes to my house frequently to ensure my health is stable and to modify my prescription when needed”. (p48) “The doctor’s regular visits to my home allow for ongoing adjustments to my treatment plan and medication”. (p2)*

Finally, dentists and physiotherapists are mentioned in smaller proportions, with only two professionals each, accounting for 3.3% of the total for each category.

*“The dentist has been visiting more frequently to clean my teeth, as they were quite dirty, and recently, the dentist from the health post has been coming to my house”. (p3) “Additionally, the physiotherapist comes here to help me walk, while mostly, it’s the doctor who assists me in getting up during their visits”. (p1)*

This analysis provides important data on the composition of the healthcare team involved in home care, highlighting the predominance of community health agents, followed by nursing technicians, nurses, and doctors, with a proportionally smaller presence of dentists and physiotherapists.

### 3.3. Considerations on Home Care

The majority of participants, equivalent to 36.6% of the total, emphasized the importance of continuity of treatment during home visits. This suggests that they value consistency and regularity in the care they receive at home. Interviewees’ statements:

*“Their visits are essential for maintaining my treatment; since I had surgery last week, the nurses come to change the dressing”. (p44) “Continuing the consultations has been beneficial for me, especially since I can’t go to the health post because I’m bedridden and unable to walk”. (p15)*

About 25.0% of participants highlighted the importance of humanized care, emphasizing the emotional aspect and the quality of the relationship with healthcare professionals during home visits.

*“The staff from the health post have been angels; I was having trouble walking, and they already arranged for a doctor to help me get up”. (p35) “They know what they are doing and always ask if they can help; you can see their care for us, you know?” (p39)*

A significant portion, representing 31.6% of participants, expressed satisfaction with the quality of the care received. This indicates that most are happy with the service provided.

*“I think it’s good; they come every week. Look, I have no complaints; in the other city I lived in, no one would come to my house, but here they come and provide great care”. (p17)*

On the other hand, a small proportion, corresponding to 6.6% of participants, expressed a desire for more frequent home visits. This suggests an opportunity to improve the frequency of visits to better meet patients’ needs.

*“I like that they come to my house, but they could come more often; they only come occasionally”. (p10) “They could come more often; the doctor stops by now and then, and it’s very quick, so I’m left with many questions”. (p20)*

This analysis provides a comprehensive view of participants’ perceptions of various aspects of home care, such as continuity of treatment, humanized care, quality of care, and frequency of visits. These data can be useful for guiding initiatives aimed at improving the provision of home healthcare services.

## 4. Discussion

The results of this study reveal important aspects regarding the demographic and socioeconomic characteristics of the beneficiaries of home care, highlighting the prevalence of women with low education levels. These factors reinforce the need for public policies that address the vulnerabilities of this population, as socioeconomic conditions directly affect access to healthcare and perceptions of service quality. Previous studies indicate that these characteristics influence how care is received and the level of patient involvement in the recovery process, suggesting that more personalized interventions may be necessary to adequately meet the needs of this population [34,35].

The role of community health workers (CHWs) as intermediaries in home care is also essential, as evidenced by their relevance in the continuity of care and in mediating interactions between healthcare professionals and patients. The presence of these professionals during weekly visits reinforces the importance of a structure that values proximity and trust in ongoing care. The literature suggests that CHWs are fundamental in ensuring effective care coordination and facilitating access to essential services, particularly in vulnerable areas [34,36].

Home care, carried out predominantly by nursing technicians, contributes to patient comfort and satisfaction, promoting biopsychosocial recovery [37]. However, a recurring concern is the frequency of visits. While home interventions meet the basic needs of patients, many report the need for more frequent follow-up. This suggests the need for adjustments in care protocols, especially for patients with more complex conditions, to ensure continuous and appropriate support is provided [34,35].

Another point worth highlighting is patients’ perception of the humanization of services. The continuity of care, combined with a close relationship with professionals, fosters the perception of a more human and attentive service. Studies point out that humanization in home care is strongly linked to improving patients’ quality of life and strengthening the bond between caregivers and the assisted community [38].

The analysis of socioeconomic factors reveals that low education levels and precarious financial situations are significant barriers to access and perceived quality of home care. Patients in more vulnerable conditions tend to require more support and follow-up, reinforcing the need for health policies that ensure equity in access to services and personalized attention [35,38].

Moreover, the challenges faced by healthcare professionals, such as work overload and limited resources, are critical points in the organization of the service. The literature highlights that the lack of adequate structure can lead to a decline in the quality of care and problems such as care fragmentation, which, in turn, directly affects patient satisfaction and health outcomes [39,40].

Humanization of care and continuity of care are fundamental elements for patient satisfaction with home healthcare services. These attributes ensure that care is consistent and tailored to the biopsychosocial needs of individuals, which, in turn, promotes greater treatment adherence and contributes positively to the recovery process [41,42].

On the other hand, care coordination is another aspect that needs to be strengthened. The literature shows that service fragmentation and the lack of integration between different levels of care harm the quality of care and can increase the vulnerability of the frailest patients [34,43,44,45].

The limitation of financial resources and the lack of investment in structure for home care are also barriers that directly affect the quality of the services offered. Financing models that prioritize humanized care and continuity of care are essential to ensure adequate support for patients in their own homes [35].

Finally, the limitations of this study include the use of a non-probabilistic sample. However, given the scarcity of research on the subject, this study significantly contributes to the understanding of the challenges and needs of home care beneficiaries, providing important data for improving health services.

### Future Perspectives

The results indicate an urgent need for policies that tackle not only the structural and coordination issues of home care services but also the socioeconomic inequalities that hinder access to quality care. An integrated approach is essential, one that considers the vulnerability conditions of patients—such as age, chronic health issues, and socioeconomic status—that often exacerbate their healthcare needs. This calls for the development of targeted programs that address immediate healthcare delivery while also confronting the underlying factors contributing to patient vulnerability. Additionally, enhancing access to quality home care necessitates engaging communities in the design and implementation of services, ensuring that interventions are tailored to the specific needs of the populations served.

Equally important is the need to provide adequate support for healthcare professionals involved in home care. This includes ensuring they receive proper training, resources, and emotional support to effectively manage the demands of their roles. Investing in professional development and providing access to mental health resources can enhance the quality of care delivered while mitigating the risk of burnout among healthcare workers. Future research should focus on identifying best practices in policy development, service delivery, and community engagement, ultimately creating a robust framework for home care that effectively addresses both patient needs and systemic inequalities.

## 5. Conclusions

The analysis of perceptions revealed that participants deeply value the continuity of treatment and the quality of care received. Home visits, consistently carried out, play an essential role in this process, providing regular follow-up and individualized support. Among the healthcare professionals, community health agents are the ones who most frequently make these visits, ensuring that patients’ needs are continuously and effectively addressed. Additionally, participants emphasized the importance of the proximity and bond created with these professionals, who often provide guidance on the correct use of medications, monitor treatment progress, and help resolve general health-related issues. This constant attention generates a sense of security and trust in the care received, distinguishing the assistance provided at their homes from that obtained in other cities or healthcare units, where follow-up was often more scarce or nonexistent.

## Figures and Tables

**Table 1:** Categories of perceptions of home care according to interviewees. Itatiba/SP, Brazil, 2024.

Aspect Evaluated	Category	Frequency (%)
Frequency of Home Care	Weekly	61.6
	Biweekly	33.4
	Monthly	5.0
Professionals Involved in Home Care	Community Health Agents	45.0
	Nursing Technicians	18.3
	Nurses	15.0
	Doctors	15.0
	Dentists and Physiotherapists	3.3
Perception of Home Care	Continuity of Treatment	36.6
	Humanized Care	25.0
	Satisfaction with Service Quality	31.6
	Desire for More Frequent Visits	6.6

## Data Availability

The raw data supporting the conclusions of this article will be made available by the authors on request.
